# Explainable AI associates ECG aging effects with increased cardiovascular risk in a longitudinal population study

**DOI:** 10.1038/s41746-024-01428-7

**Published:** 2025-01-13

**Authors:** Philip Hempel, Antônio H. Ribeiro, Marcus Vollmer, Theresa Bender, Marcus Dörr, Dagmar Krefting, Nicolai Spicher

**Affiliations:** 1 Department of Medical Informatics, University Medical Center Göttingen, Göttingen, Germany; 2 German Centre for Cardiovascular Research (DZHK), Partner Site Lower Saxony, Göttingen, Germany; 3 Department of Information Technology, Uppsala University, Uppsala, Sweden; 4 Institute of Bioinformatics, University Medicine Greifswald, Greifswald, Germany; 5 German Centre for Cardiovascular Research (DZHK), Partner Site Greifswald, Greifswald, Germany; 6 Department of Internal Medicine B, University Medicine Greifswald, Greifswald, Germany

**Keywords:** Risk factors, Diagnostic markers, Cardiovascular diseases

## Abstract

Aging affects the 12-lead electrocardiogram (ECG) and correlates with cardiovascular disease (CVD). AI-ECG models estimate aging effects as a novel biomarker but have only been evaluated on single ECGs—without utilizing longitudinal data. We validated an AI-ECG model, originally trained on Brazilian data, using a German cohort with over 20 years of follow-up, demonstrating similar performance (*r*^2^ = 0.70) to the original study (0.71). Incorporating longitudinal ECGs revealed a stronger association with cardiovascular risk, increasing the hazard ratio for mortality from 1.43 to 1.65. Moreover, aging effects were associated with higher odds ratios for atrial fibrillation, heart failure, and mortality. Using explainable AI methods revealed that the model aligns with clinical knowledge by focusing on ECG features known to reflect aging. Our study suggests that aging effects in longitudinal ECGs can be applied on population level as a novel biomarker to identify patients at risk early.

## Introduction

Aging is a universal biological process that is characterized by the gradual decline in physiological functions, increasing the vulnerability to cardiovascular diseases (CVD) and thus mortality. Therefore, advanced “chronological age,” i.e., the years passed since birth, is a major component of clinical risk scores^1–4^. In contrast, the concept of “biological age” aims to include individual aging effects^5^ based on the underlying hypothesis that aging is decelerated in a beneficial environment and accelerated in challenging conditions. Multiple biomarkers such as blood samples^6,7^ or cardiac ultrasound^8^ have been proposed to detect patients at risk for pronounced aging effects; however, they did not find their way to routine check-ups. In this paper, we study aging effects in the electrocardiogram (ECG) as a novel biomarker for cardiovascular risk including atrial fibrillation (AF), heart failure (HF), myocardial infarction (MI), and mortality.

ECG measures the heart’s electrical activity, offering clues not only about acute cardiac conditions but also the subtle changes associated with chronic diseases and aging. Changes in ECG patterns over time can reflect alterations in cardiac structure and function^9–11^. During aging, there are two main adaptations that are visible on the ECG. The first is ventricular hypertrophy, which describes a degree of cardiac remodeling^12^ necessary to compensate for the stiffness of aged vessels^13^, especially of the aortic arch. The second is fibrosis, which is the molecular consequence of remodeling in which functional tissue is replaced by connective tissue to limit dilatation by holding the enlarged myocardium together^14,15^. These adoptions manifest slowly over a long period of time and are influenced by the presence and absence of cardiovascular diseases^16,17^. This underlines the potential for serial ECGs, i.e., recordings from the same subject at different points in time, to reveal aging effects. Using serial instead of single ECGs, for instance, increases the performance in detecting HF^9^, malignant ventricular arrhythmias^18^, or MI^19^. For example, in MI, diagnostic ECG signs—associated with poor outcomes in single ECGs—when still present on follow-up ECGs were shown to increase mortality by a HR of 1.47 (95% CI: 1.10–2.13)^19^.

Recently, artificial intelligence-based electrocardiography models (AI-ECGs) became available which process 12-lead ECGs and predict a patient’s biological age^20,21^, denoted as ECG-age. The difference between the predicted ECG-age, i.e., the biological age of the myocardium, and the actual chronological age shows the potential to serve as a novel biomarker for cardiovascular risk: Patients whose ECG-age exceeded their chronological age by more than 8 years exhibited a higher risk of mortality or CVD, while those with an ECG-age at least 8 years less than their chronological age showed a reduced risk^20,21^.

Despite the reported high accuracy of the AI-ECG^20,21^ model, its decision-making process and influence on standard ECG parameters are still unclear. Here we provide a thorough analysis of aging effects by means of serial ECGs as well as long-term follow-ups for decades, contributing to novel insights into the AI-ECG mechanisms. However, our primary aim is not to predict mortality directly but to explore the use of ECG-based aging effects for subtle subclinical changes to identify patients at increased cardiovascular risk early. Overall, in this work, we (1) associate the ECG aging effects with increased cardiovascular risk in a population-based cohort study with long follow-ups, (2) investigate to what extent serial ECGs provide additional information compared with single ECGs, and (3) explain the aging effects that the AI-ECG model learned.

## Results

### Validation of AI-ECG

**Fig. 1:**
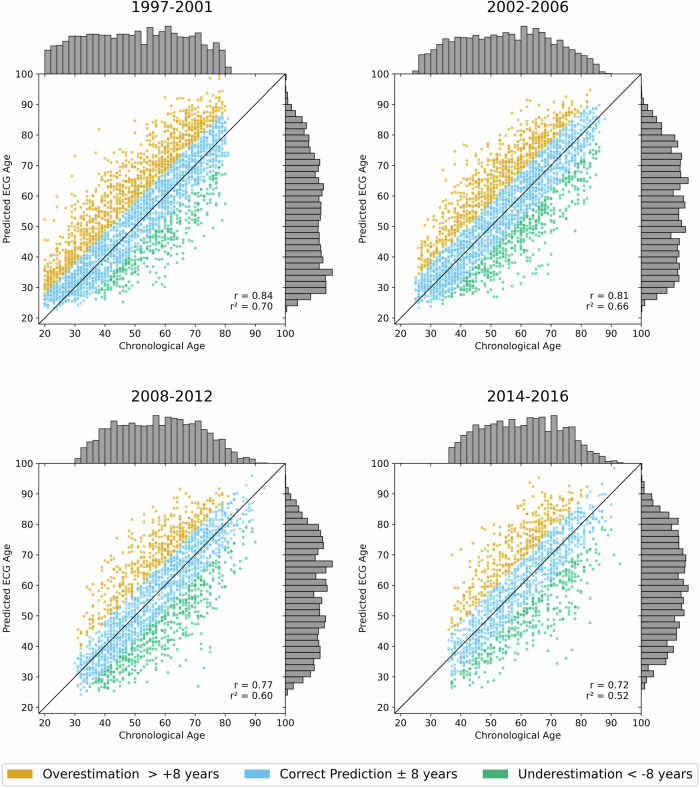
Scatter plot illustrating the age prediction of the AI-ECG. Data stems from the SHIP-START cohort study, which includes serial ECGs taken 5–6 years apart for the same individuals. The chronological age of subjects is plotted against the predicted ECG-age to assess the accuracy of the AI-ECG predictions. The color code represents the prediction accuracy: “Correct Prediction” (blue) is defined as a predicted ECG-age within ±8 years of the chronological age. “Overestimation” (orange) indicates a predicted ECG-age more than 8 years higher, while “Underestimation” (green) refers to a predicted ECG-age more than 8 years lower. Histograms display the frequency distributions of chronological and predicted ECG-ages. The correlation coefficient *r* and the coefficient of determination *r*^2^ show a decreasing trend over time.

Figure 1 shows the individual predictions of the AI-ECG in the SHIP START subjects and study phases. The differences of the chronological age at ECG recording and predicted age (*δ*_age_ = predicted age − chronological age) were computed from each ECG and classified as either “Overestimation” (*δ*_age_ > 8 years), “Correct Prediction” (−8 years ≤ *δ*_age_ ≤ 8 years), or “Underestimation” (*δ*_age_ < −8 years). The MAE of age prediction was reported in the original CODE dataset as 8.38^20^ and is 8.15 in the SHIP cohort, which leads to well-distributed group sizes. The correlation coefficient *r* and the coefficient of determination *r*^2^ are with 0.84 and 0.71 at baseline very similar to the results reported in the original publication^20^ with 0.84 and 0.70, respectively. In the follow-up, this is decreasing with increasing mean age of the cohorts which can be seen in Table 1. The baseline in “START-0” has a mean age of 49.74 years which is very similar to the mean age of the CODE dataset of 51 years. The mean age is increasing over the follow-ups until 60.12 in “START-3” with decreasing *r*^2^. Moreover, there are subtle indications of an S-curve pattern, particularly at the edges of the age distribution, where especially predictions for younger subjects (under 40 years) appear to show slightly more deviation.

**Table 1:** Summary of cohort characteristics

Cohort	Recruiting period	*n*	Female sex (%)	Mean age (years)	Mean follow-up (years)
START-0	1997–2001	4307	50.87	49.7 ± 16.4	17.6 ± 4.7
START-1	2002–2006	3299	51.82	54.4 ± 15.3	13.4 ± 3.4
START-2	2008–2012	2332	51.49	57.7 ± 13.6	8.5 ± 1.8
START-3	2014–2016	1705	53.92	60.1 ± 12.7	4.1 ± 0.8
TREND-0	2008–2011	4420	49.94	52.6 ± 15.4	8.4 ± 1.5
TREND-1	2016–2019	2484	51.64	57.2 ± 13.8	1.6 ± 0.9

The table details the recruitment periods, number of participants *n*, percentage of females, mean ages with standard deviations, and mean follow-up times for all cohorts analyzed.

**Fig. 2:**
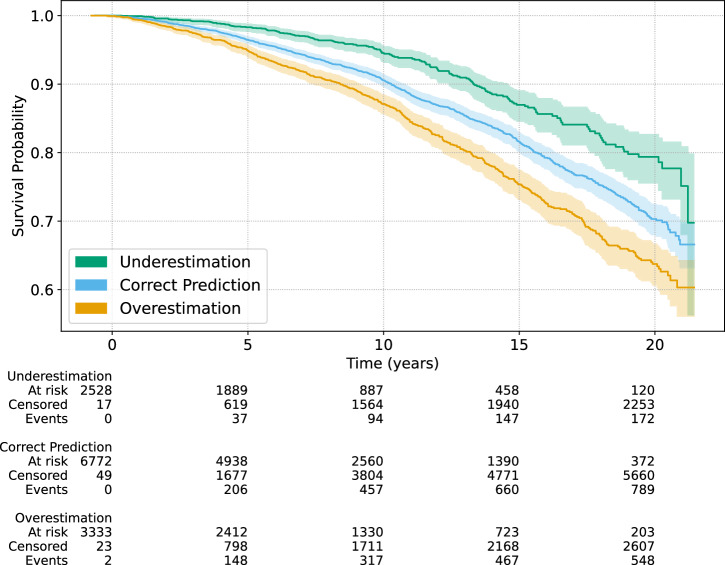
Kaplan–Meier survival estimates based on the gap between predicted ECG-age and chronological age. Results are adjusted for age and sex. Subjects with pronounced aging effects (“Overestimation”) exhibit the highest mortality rates, whereas those with reduced aging effects (“Underestimation”) exhibit the lowest.

Figure 2 depicts the survival curves of the three defined groups. The subjects classified as “Overestimation,” i.e., more pronounced aging effects, have the lowest survival rate over the whole follow-up period. Subjects classified as “Underestimation,” i.e., with less pronounced aging effects, have the highest survival rates whereas “Correct Prediction” is between both. The sex distribution differs between the groups with 40% females in “Overestimation,” 53% in “Correct Prediction,” and 66% in “Underestimation.” However, age distribution is similar with 56.80 ± 9.53 for “Overestimation,” 57.16 ± 10.25 for “Correct Prediction,” and 58.14 ± 9.92 for “Underestimation,” indicating a balanced comparison of mortality outcomes w.r.t. aging effects.

Tables 2 and 3 show the association between aging effects categories (“Overestimation” or “Underestimation,” while “Correct Prediction” serving as the reference category) and cardiovascular events, adjusted for patient demographics and relevant comorbidities.

**Table 2:** ORs for the association between cardiovascular events and AI-ECG-based aging effects

Cardiovascular event	Overestimation		Underestimation	
	OR (95% CI)	*p*-value	OR (95% CI)	*p*-value
AF diagnosed	2.72 (2.02, 3.65)	< 0.001	0.22 (0.10, 0.48)	< 0.001
Will develop AF	1.69 (1.20, 2.38)	0.003	0.37 (0.19, 0.72)	0.004
HF diagnosed	1.39 (1.20, 1.62)	< 0.001	0.78 (0.65, 0.95)	0.011
Will develop HF	1.40 (1.11, 1.78)	0.005	1.00 (0.75, 1.33)	0.997
MI diagnosed	1.27 (1.04, 1.56)	0.022	0.60 (0.44, 0.83)	0.002
Will develop MI	1.10 (0.73, 1.65)	0.653	1.24 (0.89, 1.75)	0.209
Died within study	1.47 (1.29, 1.67)	< 0.001	0.55 (0.46, 0.65)	< 0.001

The analysis includes conditions such as the diagnosis and future development (diagnosed during follow-up) of AF, HF, MI, as well as mortality (“Died within study”). Results are adjusted for chronological age, sex, diagnosed hypertension, hyperlipidemia, diabetes, and medications that affect ECG signals, including beta blockers, calcium channel blockers, and antiarrhythmics.

In Table 2, “Overestimation” was strongly linked to higher odds of AF diagnosis (2.72 (2.02–3.65), *p* < 0.001) or developing AF in future (1.69 (1.20–2.38), *p* = 0.003), while “Underestimation” significantly lowered the odds of AF diagnosis (0.22 (0.10–0.48), *p* < 0.001) or developing AF (0.37 (0.19–0.72), *p* = 0.004).

For HF, “Overestimation” increased the likelihood of both HF diagnosis (1.39 (1.20–1.62), *p* < 0.001) and future HF (1.40 (1.11–1.78), *p* = 0.005). “Underestimation,” however, was linked to reduced odds of having HF (0.78 (0.65–0.95), *p* = 0.011), while no significant effect was found for future HF (1.00 (0.75–1.33), *p* = 0.997).

In the case of MI, “Overestimation” was tied to a higher chance of a prior MI diagnosis (1.27 (1.04–1.56), *p* = 0.022), though it had no significant effect on future MI events (1.10 (0.73–1.65), *p* = 0.653). “Underestimation” corresponded to lower odds of having MI (0.60 (0.44–0.83), *p* = 0.002), with no notable impact on developing MI in future (1.24 (0.89–1.75), *p* = 0.209).

For mortality, “Overestimation” was associated with an elevated risk of death within the study (1.47 (1.29–1.67), *p* < 0.001), while “Underestimation” was linked to a decreased risk of death (0.55 (0.46–0.65), *p* < 0.001).

When separated by sex, the associations between the defined aging effect groups and cardiovascular outcomes were generally more pronounced in females compared to males (Table 3). For instance, females with “Overestimation” had notably higher odds of having AF (4.19 (2.41–7.28), *p* < 0.001) than males (2.30 (1.62–3.27), *p* < 0.001), and developing AF (2.06 (1.19–3.54), *p* = 0.009) was significant in females only. Similar trends were observed for HF and mortality, where “Overestimation” was linked to elevated odds in females, while the associations were generally weaker and non-significant in males. “Underestimation” showed a protective effect for both sexes, but this effect was also stronger in females, particularly for AF and mortality. Interestingly, MI diagnosis was significantly correlated with lower OR in males only (0.57 (0.39–0.84), *p* = 0.005).

**Table 3:** ORs for the association between cardiovascular events and AI-ECG-based aging effects, separated by sex

Cardiovascular event	Overestimation (females)			Overestimation (males)		
	OR	95% CI	*p*-value	OR	95% CI	*p*-value
AF diagnosed	4.19	(2.41, 7.28)	< 0.001	2.30	(1.62, 3.27)	< 0.001
Will develop AF	2.06	(1.19, 3.54)	0.009	1.50	(0.96, 2.32)	0.073
HF diagnosed	1.62	(1.32, 1.98)	< 0.001	1.18	(0.95, 1.47)	0.142
Will develop HF	1.49	(1.07, 2.08)	0.018	1.33	(0.96, 1.86)	0.090
MI diagnosed	1.55	(1.00, 2.39)	0.050	1.21	(0.96, 1.53)	0.108
Will develop MI	1.70	(0.99, 2.92)	0.053	1.01	(0.66, 1.55)	0.963
Died within study	1.72	(1.39, 2.14)	< 0.001	1.37	(1.17, 1.59)	< 0.001

Cardiovascular event	Underestimation (females)			Underestimation (males)		
	OR	95% CI	*p*-value	OR	95% CI	*p*-value
AF diagnosed	0.24	(0.07, 0.80)	0.021	0.22	(0.08, 0.60)	0.003
Will develop AF	0.25	(0.09, 0.72)	0.010	0.52	(0.22, 1.22)	0.133
HF diagnosed	0.78	(0.61, 0.99)	0.041	0.80	(0.57, 1.12)	0.190
Will develop HF	0.84	(0.58, 1.22)	0.363	1.33	(0.96, 1.98)	0.090
MI diagnosed	0.65	(0.38, 1.14)	0.135	0.57	(0.39, 0.84)	0.005
Will develop MI	0.96	(0.53, 1.73)	0.893	1.25	(0.71, 2.17)	0.438
Died within study	0.61	(0.47, 0.79)	< 0.001	0.50	(0.39, 0.64)	< 0.001

The analysis includes conditions such as the diagnosis and future development (diagnosed during follow-up) of AF, HF, and MI, as well as observed death. Results are presented separately for males and females, with adjustments made for chronological age, hypertension, hyperlipidemia, diabetes, and medications that affect ECG signals, including beta blockers, calcium channel blockers, and antiarrhythmics.

The results for HR regarding mortality risk are shown in Table 4. “Overestimation” was significantly associated with increased mortality risk, with a HR of 1.41 ((1.27–1.57), *p* < 0.001) after adjusting for age and sex, and an HR of 1.39 ((1.24–1.55), *p* < 0.001) with full adjustment. Conversely, “Underestimation” was associated with a reduced mortality risk, with HRs of 0.64 ((0.54–0.75), *p* < 0.001) and 0.65 ((0.55–0.76), *p* < 0.001) for the age and sex-adjusted and fully adjusted models, respectively.

**Table 4:** Association of ECG aging effects with mortality

Adjustment	Group	HR (95% CI)	*p*-value
Age and sex	Overestimation	1.41 (1.27–1.57)	< 0.001
	Underestimation	0.64 (0.54–0.75)	< 0.001
Age, sex, hypertension, hyperlipidemia diabetes, and ECG-altering medications	Overestimation	1.39 (1.24–1.55)	< 0.001
	Underestimation	0.65 (0.55–0.76)	< 0.001

This table explores the impact of further covariates on aging effects in relation to mortality, showing HRs with adjustments for age, sex, cardiovascular risk factors, and medications that affect ECG signals.

### Impact of serial ECGs

**Fig. 3:**
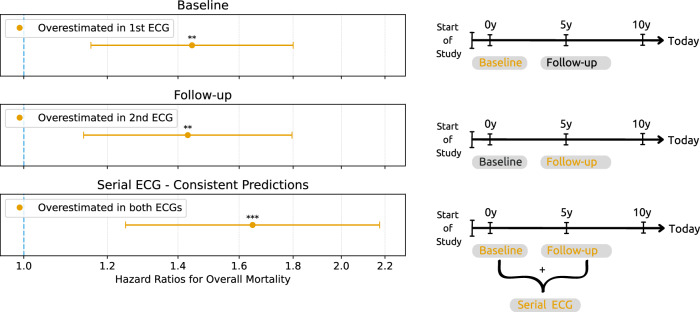
HRs for overall mortality based on serial ECG recordings. The first row presents data exclusively from a single ECG at baseline examination, illustrating initial risk association. The second row focuses on single ECG from the first follow-up examinations conducted after 5–6 years. The third row includes “Serial ECGs—Consistent Predictions” that showed “Overestimation” in both, baseline and follow-up ECGs. To analyze the influence of serial ECGs, “Follow-up” is compared to “serial ECG,” with both having a follow-up period of up to 16 years w.r.t. mortality data. “Baseline” shows the influence of a longer follow-up period w.r.t. mortality data of up to 21 years in the same individuals.

Figure 3 depicts results comparing HRs for overall mortality using different amounts of the SHIP ECG data. We compare “Overestimation” using only baseline ECGs (“baseline”), only the first follow-up (“follow-up”), and across both baseline and follow-up periods (“serial ECG - consistent prediction”). When only a single ECG is considered, the mortality risk at baseline and follow-up show a HR of 1.44 (95% CI: 1.16–1.80, *p* = 0.001) and 1.43 (1.14–1.80, *p* = 0.002), respectively. Serial ECG adds novel information to mortality prediction by increasing the HR for subjects with pronounced aging effects to 1.65 (95% CI: 1.25–2.17, *p* < 0.001). The complete results for serial ECG subgroups are shown in Table 5.

**Table 5:** Analysis of longitudinal ECG data

Model	Group	*n*	HR (95% CI)	*p*-value
Baseline	Correct prediction	1702	Reference	–
	Overestimation	874	1.44 (1.16–1.80)	0.001
	Underestimation	614	0.83 (0.59–1.18)	0.305
Follow-up	Correct prediction	1729	Reference	–
	Overestimation	849	1.43 (1.14–1.80)	0.002
	Underestimation	612	0.74 (0.53–1.02)	0.064
Serial ECG	Correct prediction, correct prediction	1165	Reference	–
	Overestimation, overestimation	551	1.65 (1.25–2.17)	< 0.001
	Correct prediction, overestimation	288	1.52 (1.04–2.21)	0.029
	Overestimation, correct prediction	311	1.34 (0.96–1.86)	0.081
	Underestimation, underestimation	351	0.68 (0.42–1.10)	0.117
	Correct prediction, underestimation	249	0.91 (0.58–1.43)	0.686
	Underestimation, correct prediction	253	1.41 (0.88–2.27)	0.154

This table presents HRs for “Overestimation” and “Underestimation” across “Baseline,” “Follow-up,” and “Serial ECG” scenarios. While “Baseline” and “Follow-up” scenarios utilize a single ECG, “Serial ECG” incorporate two ECGs recorded 5–6 years apart, simulating a longitudinal screening scenario. To investigate the influence of serial ECGs on the association between aging effects and increased cardiovascular risk, “Follow-up” is compared to “serial ECG,” both having a follow-up w.r.t. mortality data of up to 16 years.

### XAI analysis

**Fig. 4:**
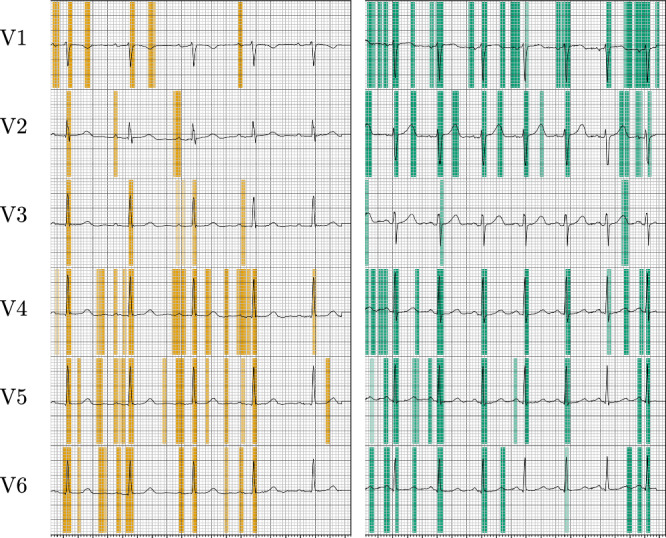
Example of two subjects that have a similar chronological age but different ECG-age. The left side depicts the precordial leads of a 51-year-old woman who is predicted as 72 years old. Relevances are highlighted in orange. The right side shows the precordial leads of a 52-year-old woman who is predicted as 41 years old. Relevances are highlighted in green. Both ECG show regular sinus rhythm whereas the first shows early R progression, which is caused by ventricular hypertrophy and partial atrial fibrosis; all findings are typical for “cor pulmonale.” The depicted grid is identical to standard ECG showing 10 mm/mV and 25 mm/s.

Regarding XAI analysis of the AI-ECG model, the precordial leads of two subjects are exemplarily plotted in Fig. 4. Pseudo-colors in green and orange depict positive relevances computed by the XAI method. On the left, XAI results for a 51-year-old woman who is predicted as being 72 years old are depicted. Relevance is mainly focused on the QRS-complex, but also on P- and T-waves. However, early R progression as a sign of right ventricular hypertrophy indicated by a dominant R wave is highlighted by relevances on lead V3. On the right, XAI results for a 52-year-old woman predicted as being 41 years old is shown. The relevances are on similar leads and waveforms compared to the ECG shown on the right, whereas the R progression is not highlighted. The full 12-lead ECGs of both examples can be seen in Supplementary Figs. 1 and 2.

**Fig. 5:**
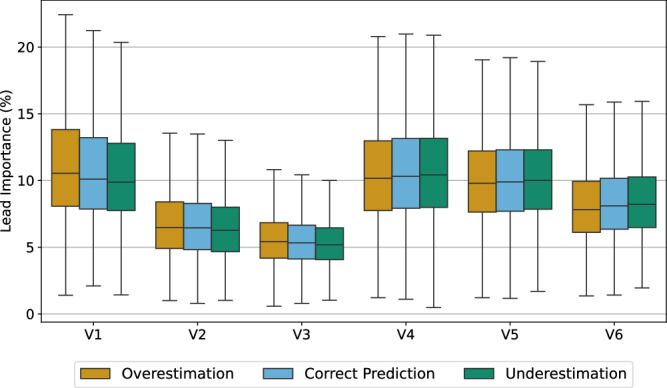
Boxplots illustrating the importance of precordial leads in the AI-ECG’s decision-making process. Results are categorized by predicted aging effects. Each boxplot shows how relevant each precordial lead was in determining whether an ECG was classified as “Overestimation,” “Correct Prediction,” or “Underestimation” based on the AI's aging predictions. This visualization helps to understand which specific leads contributed most to the AI's assessment of aging effects in the ECG recordings.

To further investigate the leads and waveforms that mostly contributed to the AI-ECG’s decision-making, lead importance is shown in Fig. 5. As can be seen, precordial leads V1 and V4 contribute mostly to the detection of the aging effect by the AI-ECG. Furthermore, there seems to be a subtle tendency for the “Overestimation” class to focus more on the right-sided leads V1-V3, whereas the “Underestimation” class leans slightly toward the left-sided leads V4-V6.

**Fig. 6:**
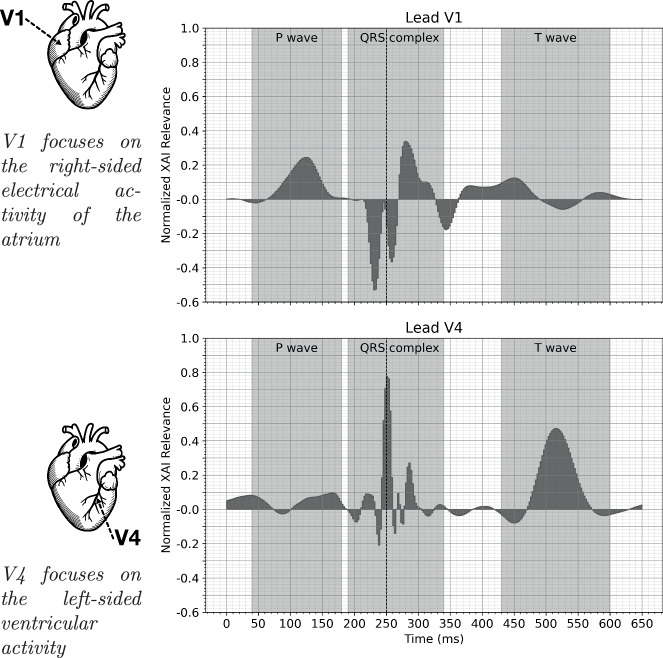
XAI relevances for leads V1 and V4 indicating the decision-making of the AI-ECG. The relevance values are normalized between −1 and 1 and aligned by the detected R peaks (dashed line) to obtain an average relevance over all subjects and heartbeats. Standard ECG parameters such as P-wave or QRS-complex are defined via physiological boundaries. Positive relevances indicating more pronounced aging effects resulting in a higher age prediction whereas negative relevances result in the prediction of younger age.

Based on the lead importance results, we selected leads V1 and V4 to investigate which part of the P-QRS-T-waveform mostly contributes to the AI-ECG’s decision-making. Figure 6 shows results for lead V1 at the top and lead V4 at the bottom. Positive and negative relevances in AI-based ECG analysis highlight parts of the waveform that influence age prediction. Positive relevances indicate regions of the ECG where aging effects are more pronounced, leading to a higher predicted age. Negative relevances, on the other hand, to regions that correspond to reduced aging effects, resulting in a younger predicted age. These relevances help to pinpoint which waveform the AI-ECG considers important in assessing biological age. In general, we observe that all parts of the ECG waveforms influence age prediction whereas both leads focus on different aspects. In lead V1, the P-wave and the QRS-complex have a major influence where the whole P-wave indicates pronounced aging effects and 30 ms before and after the R-peak indicates reduced aging effects. In contrast, the following 60 ms of the QRS-complex show positive relevances pointing to pronounced aging effects. In lead V4, one can observe mainly positive relevances for the R-peak and T-waves that indicate that these waveforms are important to detect pronounced aging effects and little emphasis on the P-wave.

**Fig. 7:**
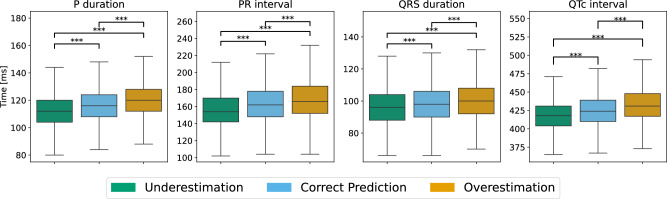
Boxplots illustrating clinical features known for aging effects. Shown are P duration, PR interval, QRS duration, and QTc interval^22^. Each feature is positively correlated with higher age groups. Boxplots display significant differences across age groups, as indicated by the asterisks (***), denoting a highly significant statistical difference (*p* < 0.001) calculated with a two-sided *t*-test.

In Fig. 7, concordance of the AI-ECG with clinical standard parameters that are known from the literature to change with the myocardial aging processes^22^ can be seen. P duration, PR interval, QRS duration and QTc interval are plotted w.r.t. the investigated groups. All parameters are highly significant differing when grouped by the AI-ECG’s detected aging effects. Despite this statistical significance, one can observe that over all boxplots, there is a high overlap, i.e., both mean and standard deviation differ only slightly. This indicates that there have to be further minor effects which are highly important for the AI-ECG model.

## Discussion

Our results indicate that the AI-ECG model is able to associate aging effects with increased mortality risk given by a HR of 1.41 (1.27–1.57, *p* < 0.001) for “Overestimation,” i.e., more pronounced aging effects, over the 21 years follow-up period of the SHIP study. “Underestimation,” i.e., reduced aging effects, results in a HR of 0.64 (0.54–0.75, *p* < 0.001). Hence, we could reproduce the findings of the initial study^20^ that showed an increase of 1.53 (1.30–1.80, *p* < 0.001) for “Overestimation” and 0.66 (0.57–0.76, *p* < 0.001) for “Underestimation” with a follow-up of 3.4 ± 1.5 years. Another work based on the population-based Framingham heart study^21^ reported an HR for mortality of 1.37 (1.25–1.50, *p* < 0.001) for “Overestimation” and HR 0.79 (95% CI: 0.73–0.86, *p* < 0.001) for “Underestimation” with a follow-up of 17 ± 8 years.

Despite mortality, the authors also reported CVDs: For AF incidence, an HR of 1.44 (1.23–1.69, *p* < 0.001) was reported for “Overestimation” and 0.89 (0.75–1.05, *p* = 0.16) for “Underestimation”^21^. While we have similar results for “Overestimation” (OR: 1.69 (1.20–2.38, *p* = 0.003)), we achieved different results for “Underestimation” (OR: 0.37 (0.19–0.72, *p* = 0.004)). The tendency for HF incidence was similar with reported HR of 1.75 (1.45–2.12, *p* < 0.001) for “Overestimation” and 0.73 (0.56–0.88, *p* = 0.002) for “Underestimation”^21^, while our OR were 1.40 (1.11–1.78, *p* = 0.005) for “Overestimation” and 0.78 (0.65–0.95, *p* = 0.011) for “Underestimation. Interestingly, the results of MI incidence were not significant in the Framingham heart study^21^ as well as in our study data. Since MI is caused by an occluded coronary vessel^23^, no significant correlation to structural heart changes like fibrosis or remodeling—that we hypothesize are learned by the AI-ECG model—seems logical. Furthermore, we found for the prevalence of MI an OR of 0.60 (0.44–0.83, *p* = 0.002) for “Underestimation,” which could indicate that structural changes of the myocardium after the MI event can be detected.

Overall, our results suggest that the AI-ECG model effectively captures fundamental aging-related changes in cardiac function that are generalizable across different geographic locations, healthcare systems, and patient populations as the ECGs were acquired in different cohorts (USA, Brazil, Germany) and stem from population and clinical studies. Previous studies have developed AI models that directly predict individual mortality from ECG data^24,25^. In contrast, our indirect approach—using aging effect as cardiovascular biomarker—is associated not only with mortality but also with the incidence of specific cardiovascular diseases, such as AF and HF. This indicates that aging effects may reflect subclinical pathologies in an early stage. Individuals at risk might benefit from early interventions before cardiovascular events like diseases or mortality occur. We hypothesize that the difference between the AI-predicted ECG-age and the chronological age captures important information about cardiovascular health that may not be fully reflected in direct mortality prediction models. However, comparing direct mortality prediction to the indirect approach using aging effects is an avenue of future work. Additionally, our analysis revealed a potential sex bias in the OR, particularly in the detection of AF incidence and mortality, suggesting an area for future investigation. Since ECG parameters are known to vary between sexes^22,26^, it may be beneficial to develop AI-ECG models that account for aging effects separately for males and females^7^ to avoid unfair predictions.

In this work, serial ECGs were used for the first time for ECG-age prediction, enabling to follow the trajectory of multiple thousand patients over time. We observed that the additional information gathered in the serial ECG improves the HR for mortality from 1.43 (95% CI: 1.16–1.80) with a single ECG from follow-up to 1.65 (95% CI: 1.25–2.17) when using both ECGs from baseline and follow-up examinations.

To gather which aging effects the AI-ECG model learned over all subjects, leads, and waveforms, we extended an established XAI workflow^27^. Analyzing individual ECG signals for their relevance distributions (Fig. 6) clearly demonstrates that relevances are aggregated at regular positions across the P-QRS-T patterns. For example, in right-sided lead V1, the AI-ECG focuses on the atrial activity (P-wave), while in left-sided lead V4, the ventricular activity (R-peak and T-wave) is of importance.

Comparing differences between leads (Fig. 5) demonstrates the importance of lead configuration. Leads V1 and V4 were of high importance while all other leads also contributed to the AI-ECG’s decision-making. Hence, the entire 12-lead ECG is important for the AI-ECG when determining aging effects. Generally, the right-sided leads V1–V3 have a tendency to more pronounced aging effects whereas the left-sided leads V4–V6 are slightly more important to predict reduced aging effects. While acknowledging the importance of all leads, we concentrated our detailed waveform analysis on leads V1 and V4 due to their specific clinical relevance. Lead V1 provides insights into atrial activity, whereas lead V4 offers information on ventricular activity. This focus allowed us to explore how the AI model captures aging effects in both atrial and ventricular regions, providing clearer insights into its predictive mechanisms. Additional analyses of all leads, including similar relevant leads I and V5, are provided in the Supplementary Figs. 3–7. Comparing our findings to established clinical ECG features known for longer durations and intervals in older individuals^22^ shows that these conventional features are not sufficient to distinguish between the groups (Fig. 7). This may be explained by the fact that the model’s decisions are based on a multifactor decision with features distributed across the heartbeats and leads as demonstrated in Fig. 6.

Regarding clinical use, our findings indicate that aging effects in ECG learned by the AI-ECG model can be linked to increased cardiovascular risk. We presume they are based on both physiological and pathological changes in cardiac structure like fibrosis and remodeling. Its decisions are based on a multifactorial process including numerous standard ECG parameters like enlarged P amplitude, prolonged PR intervals and R progression on the front wall, indicating right-sided cardiac remodeling. Since the effects are minor but significant, they can be effectively detected by the AI-ECG model. This eventually could be used to detect pronounced aging effects as subclinical indicators in common screening programs. Subsequently, cardiac remodeling could be diagnosed via cardiac ultrasound as hypertrophic myocardium, i.e., the heart muscle appears enlarged and thickened^8,12^. The gold standard to quantify cardiac fibrosis is the Late Gadolinium Enhancement, in which a contrast agent accumulates in the micro scars during a cardiac magnetic resonance imaging (MRI)^28^. However, both are not part of routine check-ups as they are expensive and time-consuming. Moreover, regularly there is no clinical consequence after this investigation since the level of remodeling and fibrosis is too advanced and cannot be reversed^29,30^. This highlights the urgent need for novel methods to quantify the level of both cardiac remodeling and fibrosis in early stages to prevent the irreversible manifestations in the myocardium^15,31–35^. Since the ECG is the most frequent diagnostic tool^36^, quantification of aging effects by AI-ECG could be a suitable tool to identify patient at risk that could benefit from additional medical attention like cardiac ultrasound or MRI.

Despite the promising results for association with increased cardiovascular risk, the current AI-ECG model has certain limitations regarding (1) age distribution, (2) sex differences, and (3) availability of serial ECG data.

First, the age range during the development of the model was unbalanced with respect to younger and older subjects since the CODE dataset is based on clinical ECG examinations from a telehealth network. Although the age range was addressed during development, effects can still be seen, especially in subject under 40 years, indicating that a more balanced age dataset could possibly further improve the robust detection of ECG-based aging effects.

Second, differences w.r.t sex were expected due to anatomical differences that are also present in other ECG-based diagnoses, such as the diagnosis of myocardial infarction^23^. Therefore, the development of sex-adjusted AI-ECG models with mitigated bias should be addressed in future work to make AI transparent and non-discriminatory^37^.

Third, regarding the follow-up period, our analysis was limited to only two serial ECGs due to the available follow-up data of the second cohort in the SHIP study. This limitation restricts the assessment of longitudinal changes in ECG patterns over time. Our study serves as a proof-of-concept highlighting the potential of serial ECGs for risk classification. Future research incorporating more ECGs across longer follow-up periods would allow for even more comprehensive insights.

As the global demographic shifts towards an older population, understanding the dynamics between aging and diseases becomes crucial. Our results underline the potential of using the standard 12-lead ECG as biomarker due to the high correlations between aging effects, disease occurrence and mortality. Our XAI results confirm that the ECG-AI model makes use of already-known ECG features—representing the underlying biological effects of cardiac remodeling—while also using a complex decision-making process with different weights for each lead. The performance of the AI-ECG model for aging effects was shown to be robust over different healthcare system (Brazil, USA, Germany) and can be improved by additional follow-up examinations (serial ECG). Since the 12-lead ECG is the most commonly used exam in regular screening programs, this method could be implemented with only minor additional effort and costs in these programs to detect cardiac remodeling early with the aim to prevent cardiovascular events before their manifestation. However, our study also shows the need for careful adjustment of the AI-ECG model, including a broader age range and different predictions depending on patient sex, to address potential bias.

## Methods

### Dataset

The Study of Health in Pomerania (SHIP) is a population-based study conducted in Western Pomerania, Germany. It encompasses two independent cohorts with follow-up examinations: SHIP-START and SHIP-TREND. The baseline examinations are designated SHIP-START-0, SHIP-TREND-0, and the follow-up examinations at 5–6 year intervals have running numbers, e.g., SHIP-START-1, SHIP-START-2. All subjects received examinations adhering to the SHIP protocol, including 12-lead ECG acquisition. Descriptive statistics for all subjects can be found in Table 1 and prevalence for CVD in Table 6. The prevalence for the CVDs was collected during the baseline and follow-up examination. Mortality data, including overall death and cardiovascular death, was extracted from the subjects’ health insurance records including a longer time period than the follow-up examinations.

**Table 6:** Disease prevalences and mortality rates across cohorts

Cohort	AF (%)	HF (%)	MI (%)	Hypertension (%)	Hyperlipidemia (%)	Mortality^a^ (%)	1-year mortality (%)
START-0	1.14	6.43	3.39	52.30	12.63	26.10	0.62
START-1	1.97	6.76	1.33	39.88	22.82	20.99	0.76
START-2	2.36	7.03	1.67	35.68	24.74	9.98	0.43
START-3	2.29	6.57	3.87	44.34	23.63	3.05	0.41
TREND-0	1.52	2.76	3.01	48.01	20.85	7.26	0.34
TREND-1	1.85	3.26	3.34	48.18	19.68	0.75	0.32

The table summarizes the prevalence of AF, HF, MI, hypertension, and hyperlipidemia, as well as overall and 1-year mortality rates for each cohort.

^a^Mortality observed until April 2019.

To further assess the influence of CVD like diabetes and ECG-altering medications on ECG signals, we extracted relevant data using “Anatomical Therapeutic Chemical” (ATC) codes (see Table 7). Specifically, we mapped each medication category to its respective ATC codes: Metformin and other oral antidiabetics (“A10BA”), Insulins (“A10A”), and antihypertensives (“C02,” “C03,” “C07,” “C08,” and “C09”). For medications impacting the ECG, we identified beta blockers (“C07AA,” e.g., metoprolol and bisoprolol), calcium channel blockers (“C08CA,” e.g., verapamil), and antiarrhythmic drugs (“C01AA,” e.g., amiodarone).

**Table 7:** Medication intake across cohorts

Cohort	Metformin (%)	Insulin (%)	Hypertensives (%)	Beta blocker (%)	Calcium channel blocker (%)	Antiarrhythmic (%)
START-0	4.60	2.30	27.77	1.53	1.72	3.85
START-1	1.53	2.12	26.47	0.29	2.91	1.26
START-2	1.63	2.05	29.95	0.12	3.07	0.55
START-3	1.55	1.80	28.59	0.13	2.89	0.33
TREND-0	1.77	1.80	31.12	0.19	2.82	0.61
TREND-1	1.59	1.38	27.02	0.09	2.67	0.35

The table provides insights into medication usage patterns within the study population.

### Ethical considerations

The SHIP studies adhered to the ethical guidelines of the 1964 Helsinki Declaration. The Ethics Committee at the University Medicine Greifswald approved the SHIP studies (approval number BB 39/08). All participants provided written informed consent before participation in the study. Data for this work was acquired via the Transfer Unit for Data and Biomaterials of the Institute of Community Medicine at the University Medicine Greifswald.

### AI-ECG model

We use a freely available pre-trained ResNet (available at: https://github.com/antonior92/ecg-age-prediction) trained on clinical ECG exams of the Brazilian TeleHealth Network of Minas Gerais^20,38,39^. The model processes 12-lead ECGs and predicts the age of the patient in an end-to-end fashion, without any intermediate steps—such as filtering or feature extraction—defined by a human. The model was trained on ECGs from 1,558,415 patients.

### Signal quality

To assess the quality of the ECG signals, we calculated the Signal-to-Noise Ratio (SNR) for each recording of the SHIP study. Following the methodology described in previous work^40^, the SNR was computed based on the ratio of signal power to noise power in the ECG frequency spectrum. We used the Fourier Transform to separate the signal frequencies (0.66 to 2.5 Hz, corresponding to 40–150 beats per minute) from the noise frequencies and expressed the SNR values on a logarithmic decibel scale.

To evaluate the impact of signal quality on our model’s predictions, we investigated the relationship between SNR and the predicted ECG-age. Pearson correlation analysis revealed no significant linear relationship, with a correlation coefficient of *r* = −0.0016 and a *p*-value of 0.857. This indicates that the AI-ECG model is robust to variations in signal quality. In a previous work, we benchmarked the impact of noise on deep learning^40^ and similarly showed that the influence of noise is minimal for this model architecture due to its ability to filter noise in the initial layers. Additionally, during the development of the AI-ECG, raw data were used, and similar observations were made regarding signal quality^20^.

In conclusion, no ECGs were excluded from our analyses due to signal quality.

### ECG aging effects and cardiovascular risk

As proposed in ref. ^20^, we use a mean absolute error (MAE) of 8 years as threshold: If the predicted ECG-age of a subject is more than 8 years higher than the real chronological age, we assume “Overestimation,” i.e., more pronounced aging effects. ECG-ages that are more than 8 years lower than the chronological age are classified as “Underestimation,” referring to reduced aging effects. To account for the current limitation of the model to older age groups, we examine a typical age range for population-wide screening programs of 40–75 years, including 12,724 ECG recordings. The lower age limit of 40 years has been set since the age group of subjects under 40 years was underrepresented during the development of the AI-ECG model^20^. Furthermore, it is anticipated that the majority of this age group will not exhibit pronounced age-related effects. The upper limit of 75 years was set since the age range of the SHIP^41^ is higher than that of the CODE dataset^20,38^, especially in the follow-up periods. Information on vital status of all SHIP study participants was requested from local health authorities in regular intervals from the time of enrollment into the study through last contact March 31, 2019. Subjects were censored at death or last contact. The number of years between examination and censoring was used as the follow-up length w.r.t. vital status. To analyze the incidence of CVDs, the prevalence was used to calculate which subjects developed AF, HF or MI between follow-up examinations.

### Serial ECG analysis

The Study of Health in Pomerania (SHIP) includes successive ECGs from the same individuals at multiple time points, offering a perspective on how ECGs change over time. We simulate a screening scenario by taking the 3190 subjects who participated in the first two examinations: Serial ECGs were defined as having exactly two ECG recordings for the same individual taken at baseline examination (“START-0,” “TREND-0”) and first follow-up examination (“START-1,” “TREND-1”). In the serial ECG cohort, the mean age is 53.89 years including 51.85% female and an overall mortality rate of 11.25%. We defined consistent predictions as those where participants were categorized into the same risk group across both their baseline and follow-up ECGs. This approach highlights stable patterns in ECG-derived predictions, reinforcing the robustness of classifications over time. To compare the impact of serial ECGs compared to single measurements, we applied three different scenarios:

1. Baseline: Using only the ECGs recorded during the initial recruitment.
2. Follow-up: Using only the first follow-up ECGs.
3. Serial ECG: Using ECGs from both time points.

To prevent label leakage, we compare only “Follow-up” directly with “Serial ECG” when investigating the influence of longitudinal data for aging effects associated with increased mortality risk. The time to mortality is adjusted for both conditions to only start at the time of the “Follow-up” examination. The single ECG at “Baseline” can be compared to “Follow-up” to investigate the association over time and the impact of longer follow-up periods w.r.t. mortality.

### XAI analysis

We extend and adapt a previously validated Explainable AI(XAI) approach^27^ using the posthoc attribution method integrated gradients^42^ to gain insight into the AI-ECG decision-making. We compute “relevance” values for each input sample of each processed ECG and visualize them on top of the standard 12-lead ECG to highlight the most relevant part for aging effects. Importance of individual leads is calculated by lead-wise addition of all relevance values and subsequent normalization across leads. To investigate which part of the P-QRS-T sequence is most important for AI-ECG decision-making, we first detect the R-peaks in the original ECG recordings using the freely available xQRS^43^ method. For robust detection, we implemented a majority vote to ensure that the R-peak is simultaneously detected in at least 7 leads with a tolerance of 10 ms; otherwise, the heartbeat is not considered. After splitting the ECGs into individual heartbeats, we extract fixed-length segments of 650 ms (250 ms before and 400 ms after each R-peak), aligning each heartbeat without altering durations. This approach maintains the PQRST fiducial points at their physiological positions within each segment.

To prevent overlap due to tachycardia or premature ventricular activity, we excluded segments where the fixed-length windows around R-peaks would overlap with adjacent segments. This exclusion ensures that each analyzed heartbeat is independent and free from interference caused by overlapping cardiac cycles. We define physiological boundaries around the R-peak for visualization (e.g., P-wave) and average the relevances lead-wise across all subjects and recordings. We selected leads V1 and V4 for detailed analysis in our XAI approach, illustrating how the AI-ECG model detects aging effects in different cardiac regions.

All XAI analyses were conducted using Python and are freely available at https://gitlab.gwdg.de/medinfpub/biosignal-processing-group/detecting-aging-effects-in-serial-ecg.

### Clinical features describing ECG aging effects

We use clinical standard ECG parameters which are known to change due to aging^22^ to compare them to the AI-ECG’s decision. The ECG parameters were taken from the SHIP dataset and were measured during the cardiological investigations, namely P duration, PR interval, QRS duration, and QTc interval. The P duration represents the time required for the atria to depolarize, whereas the PR interval refers to the delay between the atria and ventricles in the atrioventricular (AV) node. The QRS duration shows the time needed for the ventricular depolarization while the QTc interval provides the total time for depolarization and repolarization in the ventricles corrected by the heart frequency.

### Statistical analysis

To assess the association between AI-ECG-based aging effects and clinical outcomes, ORs and HRs were calculated using logistic regression and Cox proportional hazards models, respectively.

For OR calculation, logistic regression models were applied with predictors being the defined aging effect groups (“Overestimation” or “Underestimation,” with “Correct Prediction” serving as the reference category) for each clinical condition: AF prevalence (“AF diagnosed”), AF incidence (“Will Develop AF”), HF prevalence (“HF diagnosed”), HF incidence (“Will Develop HF”), MI prevalence (“MI diagnosed”), MI incidence (“Will Develop MI”), and Mortality. Models were adjusted for key confounding factors: chronological age, sex, diagnosed hypertension, hyperlipidemia, diabetes (metformin or insulin application), and medications affecting ECG signals (beta blockers, calcium channel blockers, antiarrhythmics). The full OR data are provided in Supplementary Tables 1–4.

For HRs, Cox proportional hazards models examined the relationship between the defined aging effect groups and mortality. During validation on all SHIP ECG cohorts, models included chronological age and sex, with additional adjustments for hypertension, hyperlipidemia, diabetes, and ECG-altering medications. In the smaller serial ECG cohort (3190 subjects), models were adjusted for chronological age and sex. HRs with 95% confidence intervals were reported for each model. Survival plots for both sex and medication subgroups can be seen in Supplementary Figs. 8 and 9.

Statistical significance was determined for each predictor by evaluating the *p*-values obtained from model coefficients. Additionally, two-sided *t*-tests were performed to assess differences in clinical features known to influence aging effects.

## Supplementary information

Supplementary Information

## Data Availability

The data of the SHIP study cannot be made publicly available due to the informed consent of the study participants, but it can be accessed through a data application form available at https://fvcm.med.uni-greifswald.de/ for researchers who meet the criteria for access to confidential data.

## Code availability

All scripts for analysis, including parallelization for HPC computation, and for generating the figures are freely available in Python at https://gitlab.gwdg.de/medinfpub/biosignal-processing-group/detecting-aging-effects-in-serial-ecg.
