# Supplementary material for: Explainable AI associates ECG aging effects with increased cardiovascular risk in a longitudinal population study
Source: NPJ Digit Med. 2025 Jan 13;8:25. doi: 10.1038/s41746-024-01428-7 (PMC11730300; doi:10.1038/s41746-024-01428-7)
Supplement: Supplementary file 1 — Supplementary Information [file 41746_2024_1428_MOESM1_ESM.pdf]

Supplementary Information

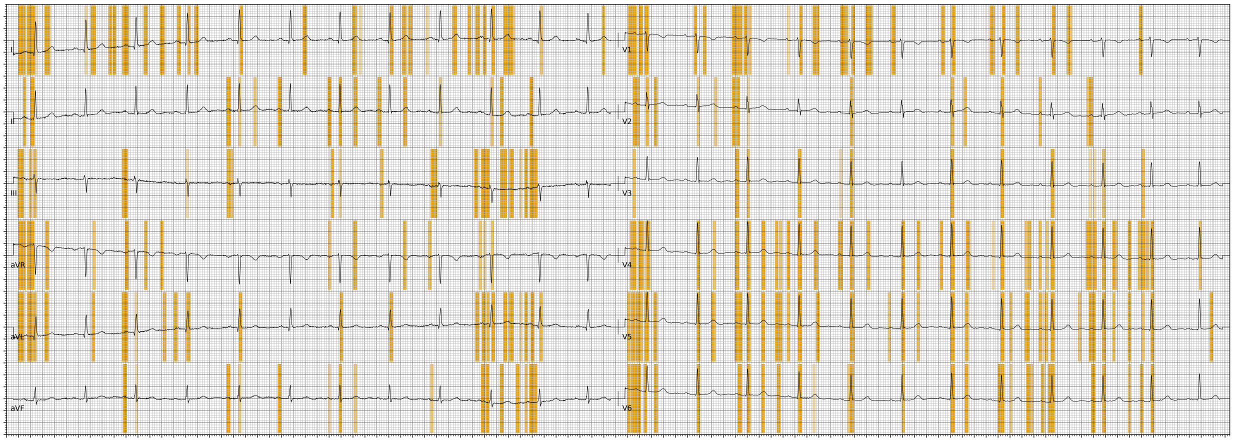

Supplementary Figure 1: **Example for "Overestimation":** 12 lead ECG of a 51 year old woman who is predicted as 72 years old.

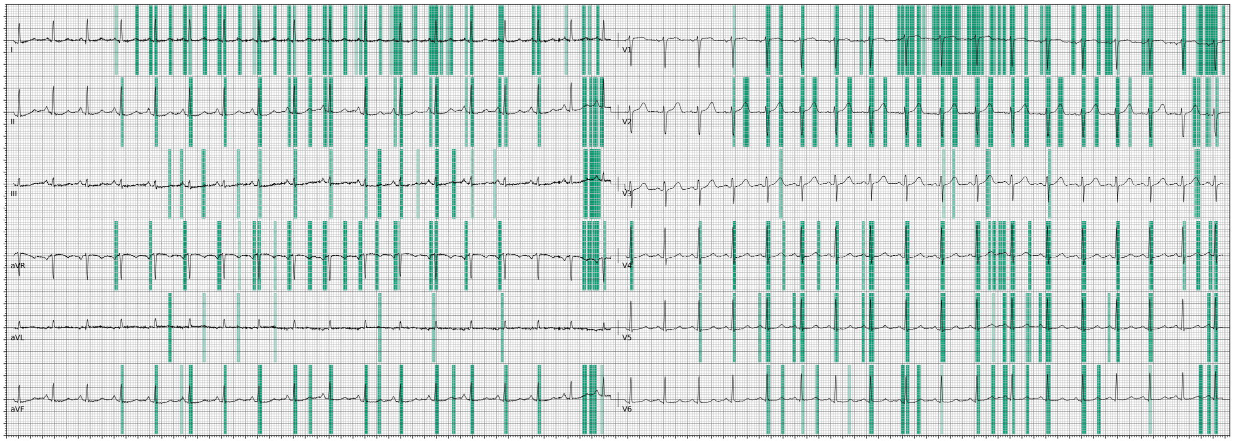

Supplementary Figure 2: **Example for "Underestimation":** 12 lead ECG of a 52 year old woman who is predicted as 41 years old.

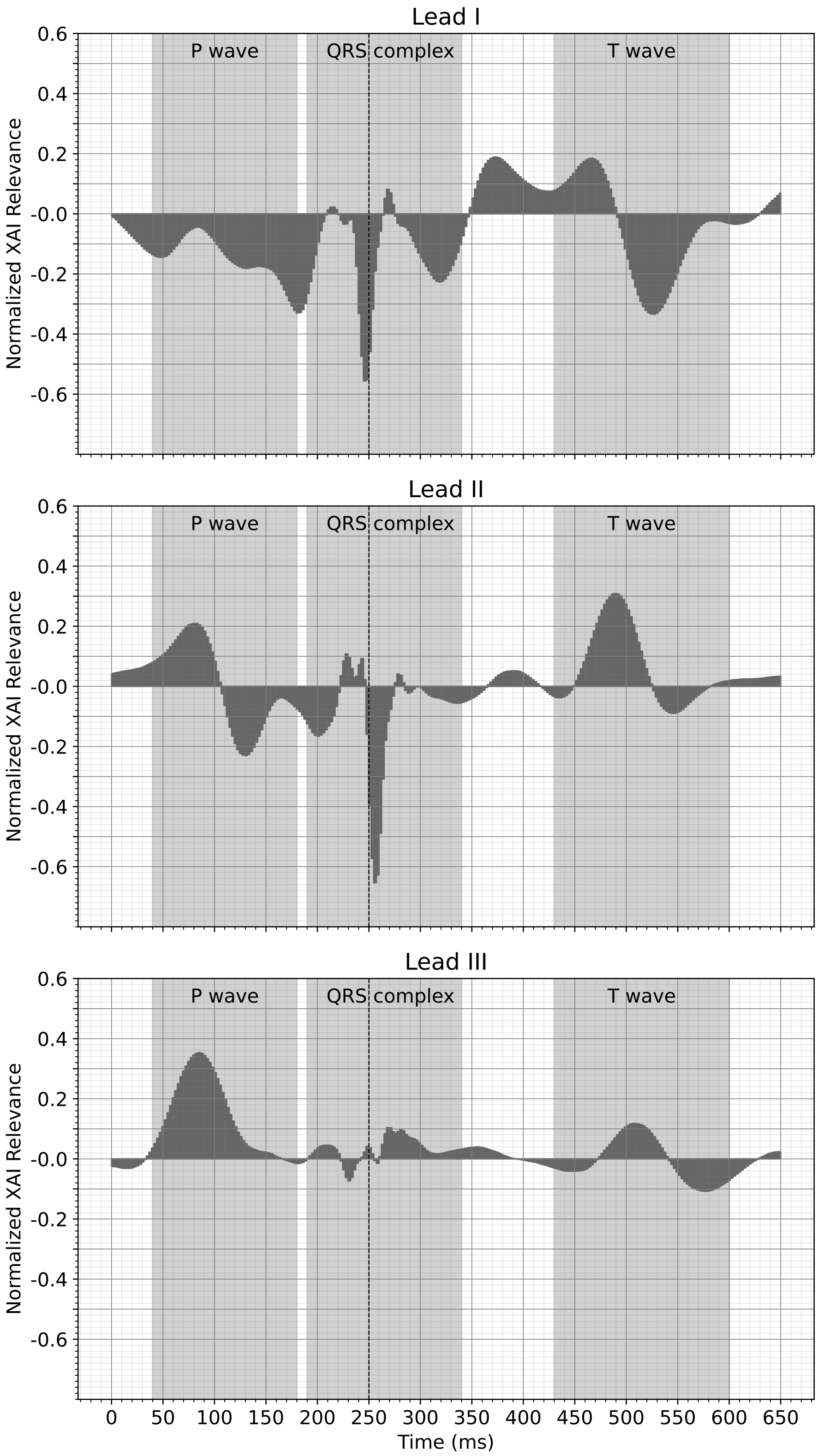

Supplementary Figure 3: **XAI relevances for Eindhoven leads:** Results for leads I, II, and III.

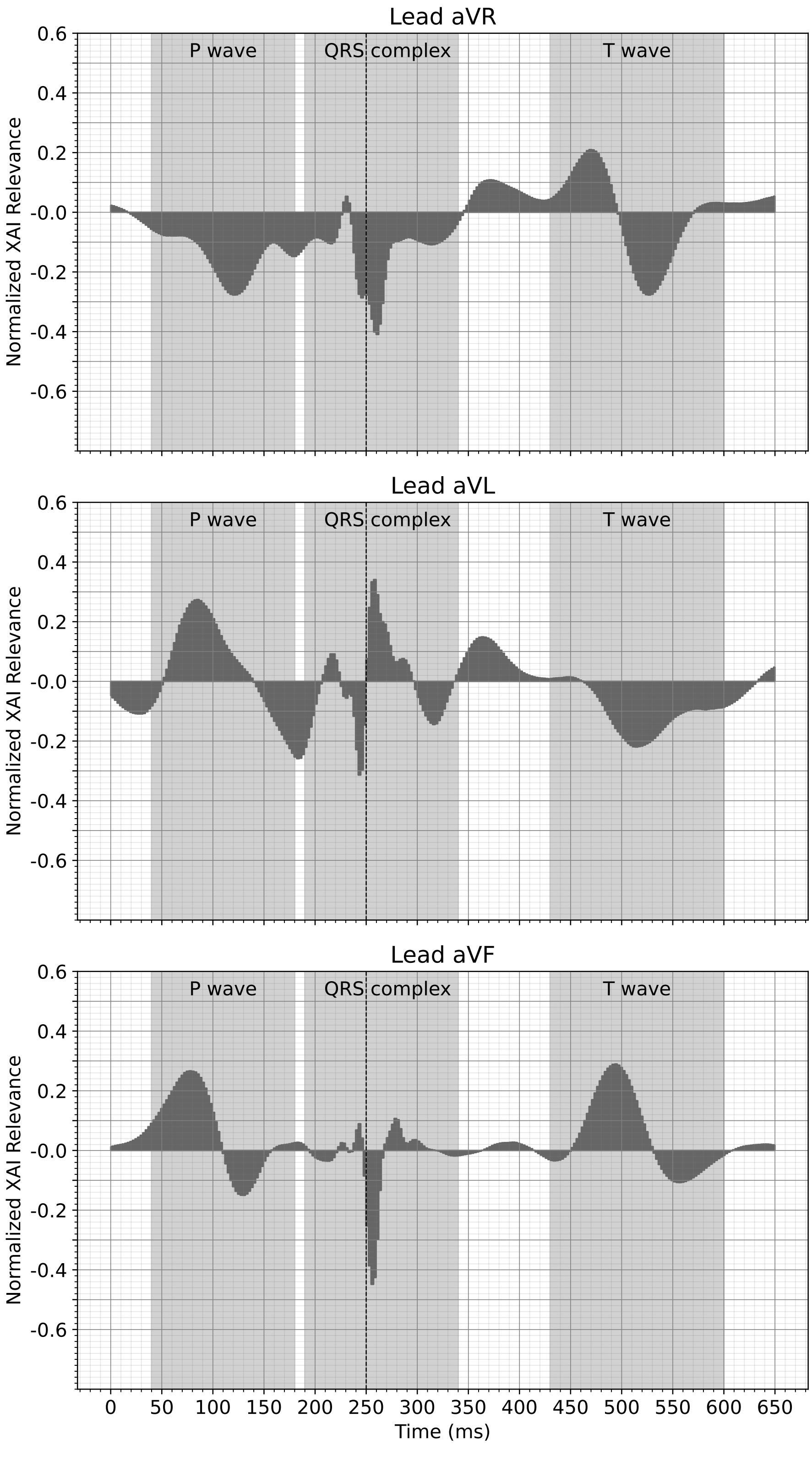

Supplementary Figure 4: **XAI relevances for Goldberger leads:** Results for leads aVR, aVL, and aVF.

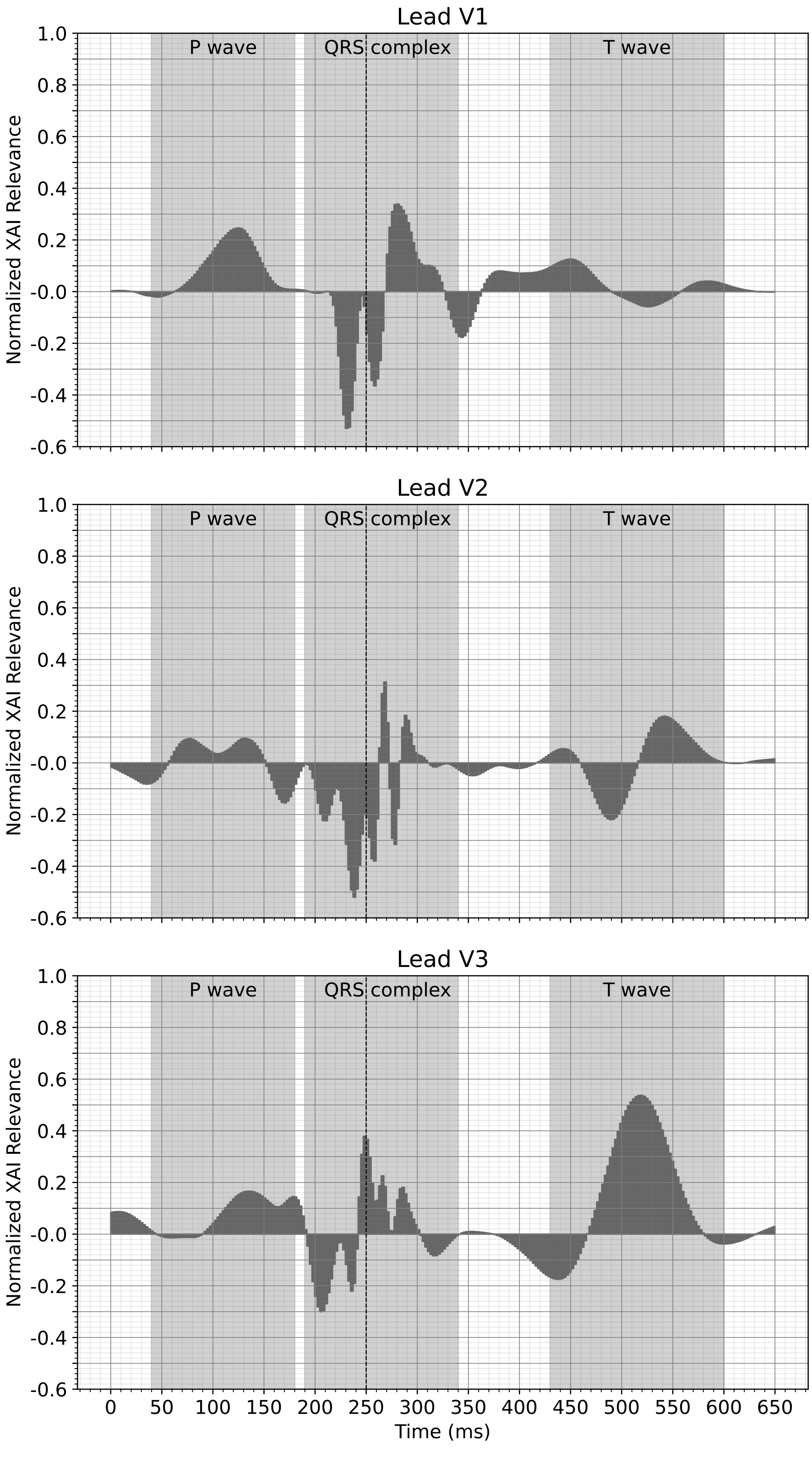

Supplementary Figure 5: **XAI relevances for anteroseptal leads:** XAI results for leads V1, V2, and V3.

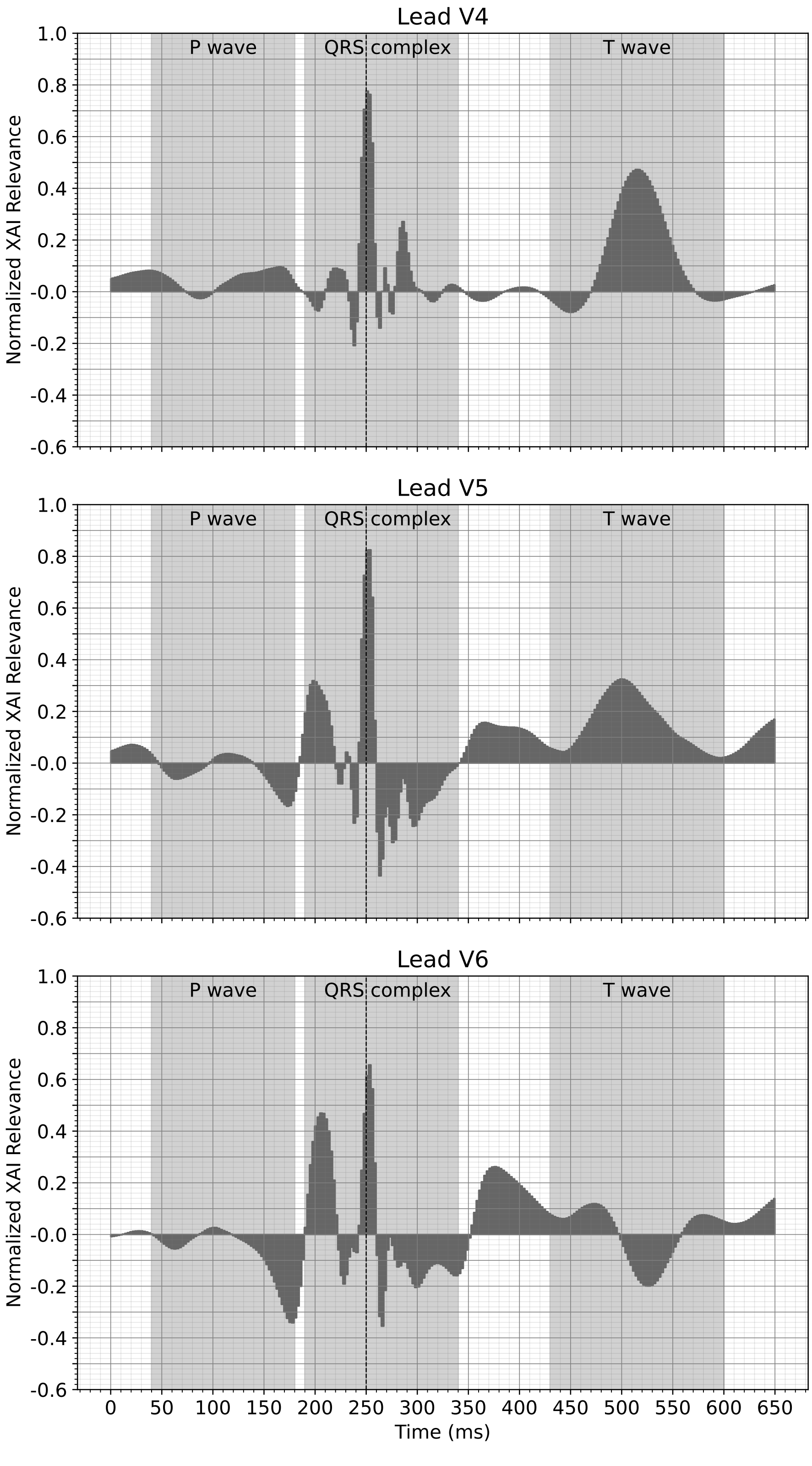

Supplementary Figure 6: **XAI relevances for lateral leads:** Results for leads V4, V5, and V6.

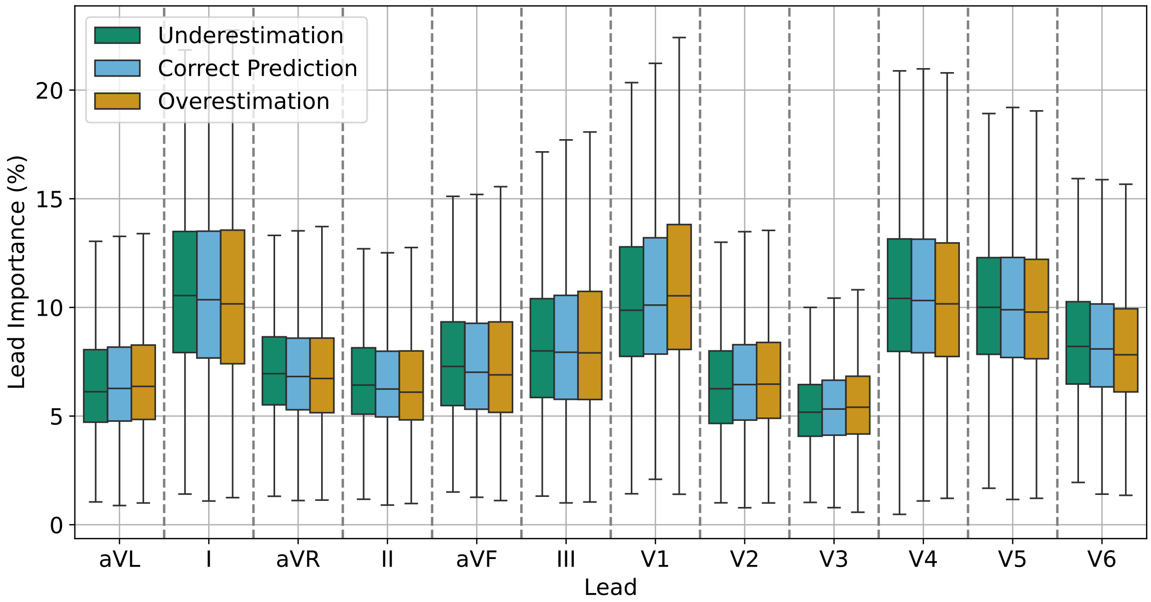

Supplementary Figure 7: **Boxplots illustrating the importance of all leads in the AI-ECG's decision-making process.** Each boxplot shows how relevant each precordial lead was in determining whether an ECG was classified as "Underestimatio", "Correct Prediction", or "Overestimation" based on the AI's aging predictions. This visualization helps to understand which specific leads contributed most to the AI's assessment of aging effects in the ECG recordings.

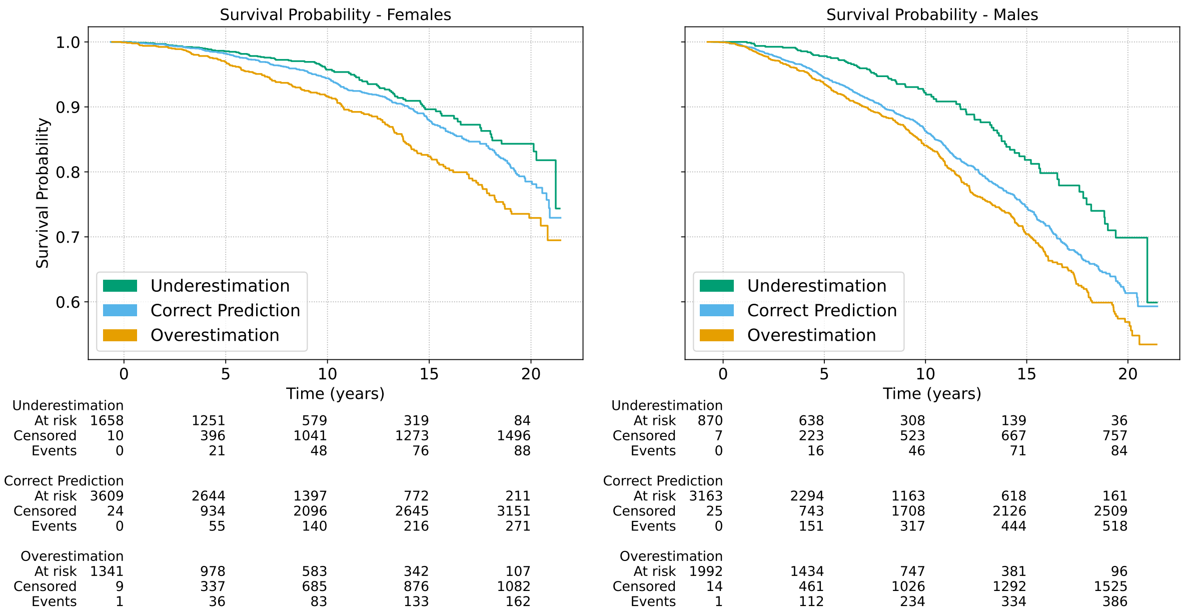

Supplementary Figure 8: **Survival for Females and Males:** Kaplan-Meier survival plots illustrating sex differences in survival probabilities across AI-based ECG risk groups.

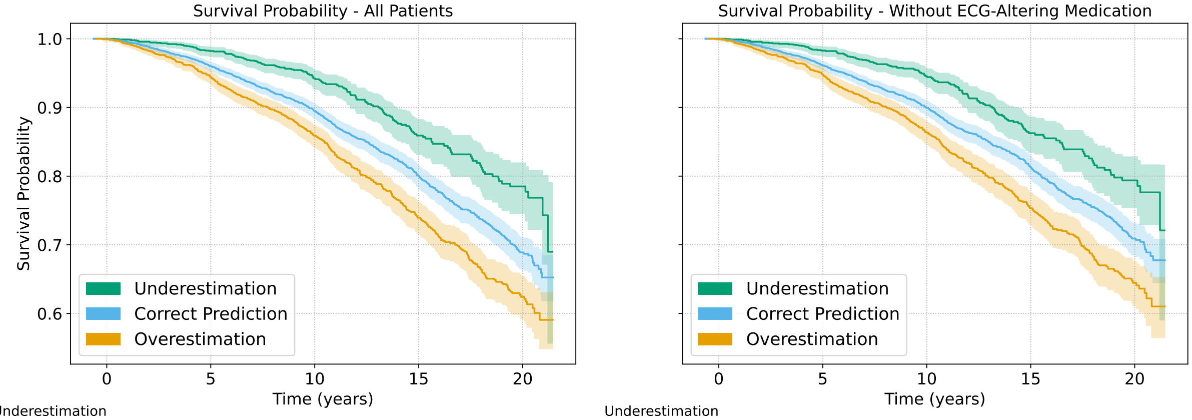

All Patients:

	0	5	10	15	20
**Underestimation**					
At risk	1938	1450	733	410	120
Censored	13	471	1141	1414	1680
Events	0	30	77	127	151
**Correct Prediction**					
At risk	5326	3930	2158	1278	372
Censored	31	1242	2786	3477	4255
Events	0	185	413	602	730
**Overestimation**					
At risk	2719	2011	1163	684	203
Censored	15	592	1282	1621	2021
Events	2	133	291	431	512

Without ECG-Altering Medication:

	0	5	10	15	20
**Underestimation**					
At risk	1875	1404	708	397	115
Censored	13	456	1109	1372	1633
Events	0	28	71	119	140
**Correct Prediction**					
At risk	5115	3767	2066	1232	360
Censored	29	1206	2701	3371	4130
Events	0	171	377	541	654
**Overestimation**					
At risk	2562	1887	1080	639	184
Censored	15	574	1236	1558	1944
Events	1	117	262	381	450

Supplementary Figure 9: **Survival for ECG Influencing Medications:** Kaplan-Meier survival curves comparing the overall survival probability between different risk groups (Underestimation, Correct Prediction, and Overestimation). The left panel shows the survival probabilities for all patients, while the right panel focuses only on patients not taking any medications known to alter ECG characteristics.

Supplementary Table 1: **Strengthening the Reporting of Observational Studies in Epidemiology:** STROBE Checklist Mapping for "Explainable AI based cardiovascular risk prediction by ECG aging effects from a longitudinal population study"

Item #	Checklist Item	Page(s)
**Title and Abstract**		
1a	Indicate the study design in the title	Title (p. 1)
1b	Provide in the abstract an informative and balanced summary of what was done and what was found	Abstract (p. 1)
**Introduction**		
2	Explain the scientific background and rationale for the investigation being reported	Introduction (pp. 2–3)
3	State specific objectives, including any prespecified hypotheses	Introduction (p. 3)
**Methods**		
4	Present key elements of study design early in the paper	Introduction (p. 3)
5	Describe the setting, locations, and relevant dates, including periods of recruitment, exposure, follow-up, and data collection	Methods: Dataset (p. 9); Table 1 (p. 17)
6a	Give the eligibility criteria, and the sources and methods of selection of participants	Methods: Dataset (p. 9)
6b	For matched studies, give matching criteria and number of exposed and unexposed	Not applicable
7	Clearly define all outcomes, exposures, predictors, potential confounders, and effect modifiers; give diagnostic criteria, if applicable	Methods: AI-ECG Model (p. 9); Statistical Analysis (pp. 11–12)
8	For each variable of interest, give data sources and details of methods of assessment (measurement)	Methods: Dataset (p. 9); AI-ECG Model (p. 9); Statistical Analysis (pp. 11–12)
9	Describe any efforts to address potential sources of bias	Methods: Statistical Analysis (pp. 11–12)
10	Explain how the study size was arrived at	Methods: Dataset (p. 9)
11	Explain how quantitative variables were handled in the analyses; if applicable, describe which groupings were chosen and why	Methods: Cardiovascular Risk Prediction (pp. 9–10)
12a	Describe all statistical methods, including those used to control for confounding	Methods: Statistical Analysis (pp. 12)
12b	Describe any methods used to examine subgroups and interactions	Methods: (pp. 10–12)
12c	Explain how missing data were addressed	Methods: (pp. 10–12)
12d	If applicable, explain how loss to follow-up was addressed	Methods: Dataset (p. 9)
12e	Describe any sensitivity analyses	Not applicable
**Results**		

Continued on next page

Supplementary Table 1 – continued from previous page

Item #	Checklist Item	Page(s)
13a	Report numbers of individuals at each stage of study	Results: Validation of AI-ECG (p. 3); Table 1 (p. 17)
13b	Give reasons for non-participation at each stage	Reasons not detailed in the manuscript
13c	Consider use of a flow diagram	Flow diagram not provided
14a	Give characteristics of study participants and information on exposures and potential confounders	Results: Table 1 (p. 17); Tables 6 and 7 (pp. 25–26)
14b	Indicate number of participants with missing data for each variable of interest	Specific numbers not specified in the manuscript
14c	Summarize follow-up time (e.g., average and total amount)	Results: Table 1 (p. 17)
15	Report numbers of outcome events or summary measures over time	Results: Tables 2–5 (pp. 19–20); Figures 2–3 (pp. 19–20)
16	Give unadjusted estimates and, if applicable, confounder-adjusted estimates and their precision	Results: Tables 2–5 (pp. 19–20)
17	Report other analyses done—e.g., analyses of subgroups and interactions, and sensitivity analyses	Results: Impact of Serial ECGs (p. 4); XAI Analysis (p. 5)
**Discussion**		
18	Summarize key results with reference to study objectives	Discussion (pp. 6–7)
19	Discuss limitations of the study, taking into account sources of potential bias or imprecision	Discussion (pp. 7–8)
20	Give a cautious overall interpretation of results considering objectives, limitations, multiplicity of analyses, results from similar studies, and other relevant evidence	Discussion (pp. 6–8)
21	Discuss the generalizability (external validity) of the study results	Discussion (pp. 6–8)
**Other**		
22	**Other Information:** Give the source of funding and the role of the funders	Acknowledgements (p. 13)

Supplementary Table 2: **Full OR data for AF incidence and prevalence:** ORs for "Overestimation" and "Underestimation" vs. "Correct Prediction" for AF and Future AF; adjusted for chronological age, sex, diagnosed hypertension, hyperlipidemia, diabetes (metformin and insulin), hypertension medication, and medication affecting ECG (beta blockers, calcium channel blockers, antiarrhythmics).

**Predictor**	**Overestimation**			**Underestimation**		
	**OR**	**95% CI**	**p-value**	**OR**	**95% CI**	**p-value**
**Diagnosed AF** Intercept	1.054	(0.818, 1.358)	0.683	0.116	(0.087, 0.154)	< 0.001
Diagnosed AF	2.715	(2.018, 3.652)	< 0.001	0.219	(0.100, 0.476)	< 0.001
Chronological Age	0.984	(0.979, 0.988)	< 0.001	1.020	(1.015, 1.025)	< 0.001
Sex	0.638	(0.586, 0.695)	< 0.001	1.583	(1.438, 1.743)	< 0.001
Diagnosed Hypertension	1.701	(1.546, 1.871)	< 0.001	0.642	(0.581, 0.713)	< 0.001
Hyperlipidemia	1.141	(1.037, 1.255)	0.007	0.956	(0.857, 1.066)	0.415
Diabetes (Metformin)	1.104	(0.805, 1.512)	0.539	1.030	(0.720, 1.473)	0.871
Diabetes (Insulin)	0.772	(0.561, 1.062)	0.112	0.692	(0.478, 1.001)	0.051
Hypertension Medication	0.904	(0.817, 0.999)	0.048	0.988	(0.886, 1.102)	0.825
Beta Blockers	1.299	(0.657, 2.568)	0.452	1.391	(0.680, 2.845)	0.366
Calcium Channel Blockers	1.106	(0.840, 1.456)	0.473	0.940	(0.689, 1.282)	0.695
Antiarrhythmics	1.060	(0.719, 1.563)	0.768	1.201	(0.801, 1.799)	0.376
**Will Develop AF**						
Intercept	0.981	(0.762, 1.261)	0.879	0.120	(0.090, 0.160)	< 0.001
Will Develop AF	1.689	(1.198, 2.381)	0.003	0.371	(0.190, 0.723)	0.004
Chronological Age	0.986	(0.981, 0.990)	< 0.001	1.019	(1.014, 1.025)	< 0.001
Sex	0.631	(0.580, 0.687)	< 0.001	1.596	(1.450, 1.757)	< 0.001
Diagnosed Hypertension	1.687	(1.534, 1.855)	< 0.001	0.645	(0.584, 0.713)	< 0.001
Hyperlipidemia	1.146	(1.042, 1.260)	0.005	0.955	(0.856, 1.065)	0.407
Diabetes (Metformin)	1.092	(0.797, 1.497)	0.583	1.026	(0.718, 1.468)	0.886
Diabetes (Insulin)	0.777	(0.565, 1.068)	0.120	0.694	(0.480, 1.004)	0.053
Hypertension Medication	0.909	(0.822, 1.005)	0.062	0.981	(0.879, 1.094)	0.729
Beta Blockers	1.236	(0.625, 2.445)	0.542	1.474	(0.718, 3.026)	0.290
Calcium Channel Blockers	1.097	(0.834, 1.445)	0.508	0.940	(0.689, 1.281)	0.694
Antiarrhythmics	1.045	(0.709, 1.540)	0.825	1.203	(0.802, 1.803)	0.371

Supplementary Table 3: **Full OR data for HF incidence and prevalence:** ORs for "Overestimation" and "Underestimation" vs. "Correct Prediction" for HF; adjusted for chronological age, sex, diagnosed hypertension, hyperlipidemia, diabetes (metformin and insulin), hypertension medication, and medication affecting ECG (beta blockers, calcium channel blockers, antiarrhythmics).

**Predictor**	**Overestimation**			**Underestimation**		
	**OR**	**95% CI**	**p-value**	**OR**	**95% CI**	**p-value**
**Diagnosed HF**						
Intercept	1.131	(0.874, 1.463)	0.348	0.103	(0.077, 0.138)	< 0.001
Diagnosed HF	1.393	(1.201, 1.617)	< 0.001	0.783	(0.648, 0.947)	0.011
Chronological Age	0.985	(0.981, 0.990)	< 0.001	1.019	(1.014, 1.025)	< 0.001
Sex	0.623	(0.572, 0.679)	< 0.001	1.608	(1.461, 1.770)	< 0.001
Diagnosed Hypertension	1.683	(1.531, 1.851)	< 0.001	0.645	(0.584, 0.713)	< 0.001
Hyperlipidemia	1.120	(1.017, 1.232)	0.021	0.972	(0.871, 1.084)	0.608
Diabetes (Metformin)	1.083	(0.790, 1.484)	0.620	1.035	(0.724, 1.480)	0.850
Diabetes (Insulin)	0.780	(0.567, 1.074)	0.128	0.686	(0.474, 0.992)	0.045
Hypertension Medication	0.910	(0.823, 1.006)	0.065	0.984	(0.883, 1.098)	0.778
Beta Blockers	1.270	(0.642, 2.513)	0.492	1.398	(0.683, 2.862)	0.359
Calcium Channel Blockers	1.093	(0.830, 1.439)	0.526	0.943	(0.692, 1.286)	0.711
Antiarrhythmics	1.025	(0.694, 1.512)	0.903	1.220	(0.814, 1.828)	0.336
**Will Develop HF**						
Intercept	1.131	(0.874, 1.463)	0.348	0.122	(0.092, 0.163)	< 0.001
Will Develop HF	1.404	(1.110, 1.776)	0.005	0.783	(0.648, 0.947)	0.012
Chronological Age	0.985	(0.981, 0.990)	< 0.001	1.019	(1.014, 1.025)	< 0.001
Sex	0.623	(0.572, 0.679)	< 0.001	1.608	(1.461, 1.770)	< 0.001
Diagnosed Hypertension	1.683	(1.531, 1.851)	< 0.001	0.645	(0.584, 0.713)	< 0.001
Hyperlipidemia	1.120	(1.017, 1.232)	0.021	0.972	(0.871, 1.084)	0.608
Diabetes (Metformin)	1.083	(0.790, 1.484)	0.620	1.035	(0.724, 1.480)	0.850
Diabetes (Insulin)	0.780	(0.567, 1.074)	0.128	0.686	(0.474, 0.992)	0.045
Hypertension Medication	0.910	(0.823, 1.006)	0.065	0.984	(0.883, 1.098)	0.778
Beta Blockers	1.270	(0.642, 2.513)	0.492	1.398	(0.683, 2.862)	0.359
Calcium Channel Blockers	1.093	(0.830, 1.439)	0.526	0.943	(0.692, 1.286)	0.711
Antiarrhythmics	1.025	(0.694, 1.512)	0.903	1.220	(0.814, 1.828)	0.336

Supplementary Table 4: **Full OR data for MI incidence and prevalence:** ORs for "Overestimation" and "Underestimation" vs. "Correct Prediction"; adjusted for chronological age, sex, diagnosed hypertension, hyperlipidemia, diabetes (metformin and insulin), hypertension medication, and medication affecting ECG (beta blockers, calcium channel blockers, antiarrhythmics).

**Predictor**	**Overestimation**			**Underestimation**		
	**OR**	**95% CI**	**p-value**	**OR**	**95% CI**	**p-value**
**Diagnosed MI**						
Intercept	1.131	(0.874, 1.463)	0.348	0.103	(0.077, 0.138)	< 0.001
Diagnosed MI	1.270	(1.035, 1.563)	0.022	0.590	(0.430, 0.820)	0.002
Chronological Age	0.982	(0.977, 0.987)	< 0.001	1.023	(1.018, 1.029)	< 0.001
Sex	0.648	(0.595, 0.707)	< 0.001	1.610	(1.440, 1.800)	< 0.001
Diagnosed Hypertension	1.680	(1.527, 1.848)	< 0.001	0.690	(0.610, 0.780)	< 0.001
Hyperlipidemia	1.165	(1.059, 1.282)	0.002	0.970	(0.860, 1.090)	0.632
Diabetes (Metformin)	1.095	(0.799, 1.501)	0.572	0.610	(0.390, 0.950)	0.030
Diabetes (Insulin)	1.900	(1.360, 2.660)	< 0.001	0.660	(0.370, 1.180)	0.163
Hypertension Medication	1.020	(0.920, 1.140)	0.714	1.000	(0.870, 1.140)	0.976
Beta Blockers	1.270	(0.643, 2.514)	0.491	0.780	(0.350, 1.730)	0.533
Calcium Channel Blockers	1.092	(0.829, 1.437)	0.526	0.910	(0.630, 1.320)	0.630
Antiarrhythmics	2.070	(1.390, 3.080)	< 0.001	0.820	(0.440, 1.490)	0.511
**Will Develop MI**						
Intercept	0.960	(0.747, 1.235)	0.755	0.122	(0.092, 0.163)	< 0.001
Will Develop MI	1.243	(0.885, 1.746)	0.209	1.097	(0.732, 1.645)	0.653
Chronological Age	0.986	(0.981, 0.990)	< 0.001	1.019	(1.014, 1.024)	< 0.001
Sex	0.631	(0.580, 0.687)	< 0.001	1.601	(1.453, 1.762)	< 0.001
Diagnosed Hypertension	1.691	(1.538, 1.860)	< 0.001	0.642	(0.581, 0.710)	< 0.001
Hyperlipidemia	1.141	(1.037, 1.255)	0.007	0.960	(0.861, 1.070)	0.461
Diabetes (Metformin)	1.095	(0.799, 1.500)	0.573	0.691	(0.478, 0.999)	0.050
Diabetes (Insulin)	0.781	(0.568, 1.074)	0.129	0.640	(0.360, 1.140)	0.144
Hypertension Medication	1.020	(0.920, 1.130)	0.773	1.000	(0.870, 1.130)	0.873
Beta Blockers	1.267	(0.641, 2.504)	0.493	1.410	(0.690, 2.880)	0.348
Calcium Channel Blockers	1.097	(0.834, 1.444)	0.508	0.939	(0.688, 1.280)	0.675
Antiarrhythmics	2.110	(1.420, 3.140)	< 0.001	1.200	(0.801, 1.800)	0.372

Supplementary Table 5: **Full OR data for mortality:**ORs for "Overestimation" and "Underestimation" vs. "Correct Prediction" for Mortality During Follow-Up; adjusted for chronological age, sex, diagnosed hypertension, hyperlipidemia, diabetes (metformin and insulin), hypertension medication, and medication affecting ECG (beta blockers, calcium channel blockers, antiarrhythmics).

**Predictor**	**Overestimation**			**Underestimation**		
	**OR**	**95% CI**	**p-value**	**OR**	**95% CI**	**p-value**
**Died within Study**						
Intercept	1.131	(0.874, 1.463)	0.348	0.103	(0.077, 0.138)	< 0.001
Died within Study	1.469	(1.295, 1.666)	< 0.001	0.548	(0.459, 0.654)	< 0.001
Chronological Age	0.982	(0.977, 0.987)	< 0.001	1.023	(1.018, 1.029)	< 0.001
Sex	0.648	(0.595, 0.707)	< 0.001	1.543	(1.401, 1.699)	< 0.001
Diagnosed Hypertension	1.680	(1.527, 1.848)	< 0.001	0.652	(0.590, 0.720)	< 0.001
Hyperlipidemia	1.165	(1.059, 1.282)	0.002	0.933	(0.836, 1.041)	0.212
Diabetes (Metformin)	1.095	(0.799, 1.501)	0.572	1.035	(0.723, 1.481)	0.850
Diabetes (Insulin)	0.778	(0.565, 1.070)	0.122	0.684	(0.472, 0.990)	0.044
Hypertension Medication	0.911	(0.824, 1.008)	0.071	0.981	(0.879, 1.094)	0.729
Beta Blockers	1.271	(0.643, 2.514)	0.491	1.445	(0.705, 2.963)	0.315
Calcium Channel Blockers	1.092	(0.829, 1.437)	0.531	0.949	(0.695, 1.294)	0.740
Antiarrhythmics	1.017	(0.689, 1.502)	0.932	1.234	(0.822, 1.852)	0.311
